# Effect of glymphatic system function on cognitive function in patients with chronic kidney disease

**DOI:** 10.3389/fneur.2024.1480536

**Published:** 2024-09-20

**Authors:** Junghae Ko, Bong Soo Park, Chang Min Heo, Jiyae Yi, Dong Ah Lee, Kang Min Park

**Affiliations:** ^1^Department of Internal Medicine, Haeundae Paik Hospital, Inje University College of Medicine, Busan, Republic of Korea; ^2^Department of Neurology, Haeundae Paik Hospital, Inje University College of Medicine, Busan, Republic of Korea

**Keywords:** chronic kidney disease, diffusion tensor imaging, glymphatic system, cognitive function, renal failure

## Abstract

**Objectives:**

Studies have recently shown an alteration of the structural connectivity and a dysfunctional glymphatic system in patients with chronic kidney disease (CKD). In this study, we aimed to investigate the effects of the structural connectivity and glymphatic system on the cognitive function of patients with CKD.

**Methods:**

We prospectively enrolled patients with CKD and healthy controls. The CKD group was divided into two regarding their cognitive function. All patients received brain magnetic resonance imaging, including diffusion tensor imaging (DTI). We calculated the measures of structural connectivity and diffusion tensor image analysis along the perivascular space (DTI-ALPS) index, a neuroimaging marker of the glymphatic system function, and compared the indices between groups.

**Results:**

The mean clustering coefficient, local efficiency, and small-worldness index in patients with CKD were lower than those in healthy controls (0.125 ± 0.056 vs. 0.167 ± 0.082, *p* = 0.008; 1.191 ± 0.183 vs. 1.525 ± 0.651, *p* = 0.002; 0.090 ± 0.043 vs. 0.143 ± 0.102, *p* = 0.003; respectively). The DTI-ALPS index was lower in patients with CKD than in healthy controls (1.436 vs. 1.632, *p* < 0.001). Additionally, the DTI-ALPS index differed significantly between CKD patients with and without cognitive impairment. Notably, this index was lower in patients with CKD and cognitive impairment than in patients without cognitive impairment (1.338 vs. 1.494, *p* = 0.031). However, there were no differences of the structural connectivity between CKD patients with and without cognitive impairment.

**Conclusion:**

We found lower DTI-ALPS index in patients with CKD, which could be related with glymphatic system dysfunction. Moreover, those with cognitive impairment in the CKD group had a lower index than those without, indicating a link between the glymphatic system function and cognitive function.

## 1 Introduction

Chronic kidney disease (CKD) is characterized by a steady decline in glomerular filtration rate (GFR) or observable kidney damage lasting for ≥3 months (1). The global prevalence of CKD is substantial and has recently increased. Currently, approximately 9–10% of the global population is affected (1, 2). Furthermore, neurological complications in CKD are varied and common, including central nervous system disorders, such as stroke, cognitive impairment, and encephalopathy, and peripheral nervous system disorders, such as peripheral neuropathies and autonomic dysfunction (3). As per recent reports, individuals at all stages of CKD are at a greater risk of developing dementia and cognitive impairment than those without CKD (4, 5). The brain and kidney injury have a strong pathophysiologic connection, due to the common vulnerability to vascular damage (6). In addition to common traditional cerebrovascular risk factors, chronic inflammation, oxidative stress, hypercoagulable states, and uremic toxin accumulation may also explain this relationship (4, 7).

The glymphatic system is a recently discovered, highly organized cerebrospinal fluid (CSF) transport system in the brain, responsible for waste product removal and homeostasis, similar to the peripheral lymphatic system (8–11). CSF enters the periarterial space from the subarachnoid space, through channels formed by the vascular endfeet of astrocytes, which contain aquaporin-4 (AQP4) water channels. It then mixes with the interstitial fluid, which contains metabolic waste products, such as proteins. Thereafter, the mixture is transported out of the brain through perivenous spaces, increasing the removal of waste from the brain (9–12). Recently, studies have shown the relationship between glymphatic system dysfunction and the development of neurological diseases. Additionally, several studies have reported glymphatic system dysfunction in patients with stroke, Alzheimer’s disease, epilepsy, and normal pressure hydrocephalus (13–16). Alterations in the glymphatic system function in patients with CKD have also been reported (8, 17–19).

Notably, several neuroimaging methods have been used to measure glymphatic system function in humans, including tracer-based studies using contrast agents and phase-contrast magnetic resonance imaging (MRI). However, these methods have limitations, including invasiveness, technical complexity, and contact exposure (9, 20). A diffusion tensor image analysis along the perivascular space (DTI-ALPS) is another advanced, non-invasive method that assesses glymphatic system function. It evaluates water molecules’ movement along the perivascular space by using diffusion tensor imaging (DTI) to relative measure of diffusivity (15, 20). Reportedly, the DTI-ALPS index has been related with glymphatic system function, which is demonstrated in various neurological disorders (13–15). Furthermore, patients with CKD exhibit lower DTI-ALPS index than that in healthy controls, which may be related with glymphatic system dysfunction (8, 17, 18). However, these studies lacked research on the relationship between cognitive function and the glymphatic system in patients with CKD.

Recent studies have been conducted to compare structural connectivity in patients with CKD with the normal group using DTI and graph theoretical analysis, and the results have proven that integration and segregation are different between the groups (21, 22).

In this study, we aimed to demonstrate changes in the structural connectivity and DTI-ALPS index in patients with CKD regarding the presence or absence of cognitive impairment. We hypothesized that there is a significant association between cognitive function and DTI-ALPS index in patients with CKD.

## 2 Methods

### 2.1 Participants: patients with CKD and healthy controls

This study was approved by our institutional review board. On approval, we prospectively enrolled patients with CKD and healthy controls at our hospital. These patients showed normal brain MRI without structural lesions or lacunar stroke. The criteria for CKD were an estimated GFR (eGFR) calculated by the CKD-EPI Creatinine Equation of more than 15 mL/min/1.73 m^2^. Patients with CKD had no history of neurological disorders and healthy controls had no history of any medical or neurological disorders.

We evaluated cognitive function in these patients using the Korean version of the Montreal Cognitive Assessment (K-MoCA). They were divided into two groups based on their cognitive function. Consequently, a K-MoCA score of ≥23 points was defined as no cognitive impairment; however, ≤22 points indicated cognitive impairment (23).

As the inclusion criteria, we enrolled 56 patients with CKD and 38 healthy controls. Age and sex did not differ between both groups. Of the 56 patients with CKD, 21 had cognitive impairment and 35 did not. Of the 56 patients with CKD, 28 patients had type 2 diabetes mellitus (T2DM), and 45 patients had hypertension.

### 2.2 Diffusion tensor imaging acquisition and calculation of structural connectivity and the DTI-ALPS index

All patients received brain MRI using the same 3-tesla MRI scanner with a 32-channel head coil (AchievaTx, Philips Healthcare). The MR sequences included three-dimensional fluid-attenuated inversion recovery (FLAIR), axial T2-weighted imaging, three-dimensional T1-weighted imaging, and DTI. The FLAIR and T2-weighted imaging were performed to check for structural lesions in the brain, and T1-weighted imaging and DTI were used to analyze structural connectivity and DTI-ALPS index in this study. Three-dimensional T1-weighted images were obtained using a turbo-field echo sequence with the following parameters: TI = 1,300 ms, repetition time/echo time (TR/TE) = 8.6/3.96 ms, flip angle (FA) = 8°, and 1 mm^3^ isotropic voxel size. The DTI acquisition parameters were as follows: 32 different diffusion directions, repetition time/echo time = 8,620/85 ms, flip angle = 90°, slice thickness = 2.25 mm, acquisition matrix = 120 × 120, field of view = 240 × 240 mm^2^, and *b*-value = 1,000 s/mm^2^.

The structural connectivity and DTI-ALPS index was calculated based on DTI using DSI Studio. We preprocessed the DTI using DSI Studio. Initially, we opened source DTI images and created a mask with following steps: thresholding, smoothing, and defragment. We also conducted preprocessing with FSL’s top-up and eddy to handle susceptibility artifacts and eddy current distortion. Then, we generated one fiber orientation per voxel and associated anisotropy and diffusivity measure with generalized q-sampling imaging reconstruction to T1-wighted space. This approach handles the individual difference in brain parcellation, but fiber tracking is done in the native space. The diffusion data is reconstructed to subject’s T1-wighted space so that all following analysis is in the T1-weighted coordinates.

Then, we calculated the network measures of structural connectivity in the participants. We performed fiber tacking with seeding the whole brain region. A deterministic fiber tracking algorithm was used. Finally, we performed spatial normalization to ensure that the built-in parcellation atlas with AAL3 (24) was registered with the subject data by applying tensor-based nonlinear registration. Network measures were calculated with graph theory. The connectivity matrix was normalized to choose the weighed measures, such that the maximum value of the matrix was one. The structural connectivity was assessed with measures such as mean clustering coefficient, global efficiency, local efficiency, characteristic path length, and small-worldness index (25–27).

**Figure 1:**
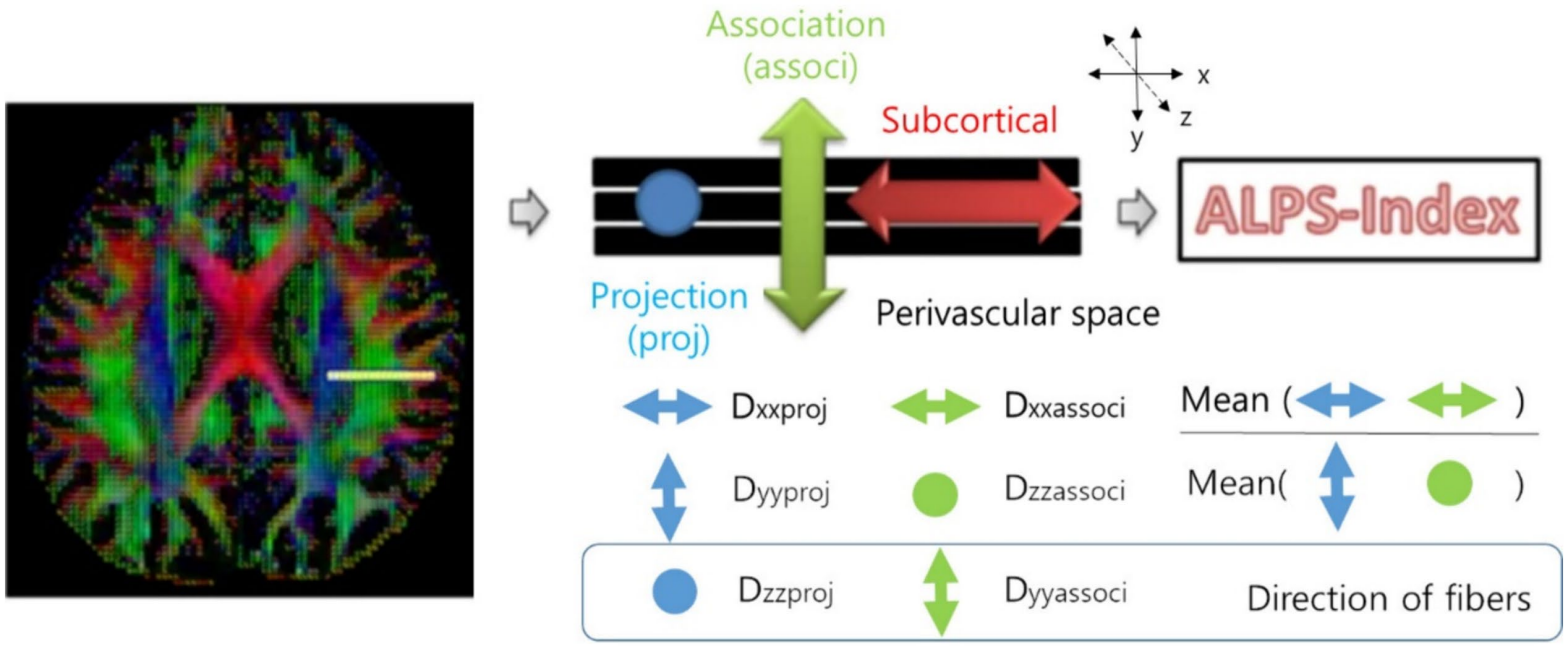
Calculation of the DTI-ALPS index (15). DTI-ALPS index: diffusion tensor image analysis along the perivascular space index.

To obtain DTI-ALPS index, we drew a 5 mm diameter spherical region of interest, in which the lateral projections of the medullary veins were traced orthogonally to the primary diffusion directions in the left hemisphere (15). Next, we obtained the fiber orientation and diffusivities in three directions along the *x*-, *y*-, and *z*-axes as the voxel levels in the region of interest. Among the various voxels, one was selected for each fiber on the same *x*-axis (projection, association, and subcortical fibers) that presented the maximum orientation in each fiber. The DTI-ALPS index was calculated using the DTI-ALPS index formula (Figure 1) (15).

$\begin{array}{l} DTI-\mathrm{ALPS index}=\mathrm{mean}\;\left(D\mathrm{xproj},D\mathrm{xassoc}\right)\\ \;/\mathrm{mean}\;\left(D\mathrm{yproj},D\mathrm{zassoc}\right)\text{.} \end{array}$

D*xproj*: diffusivity along the *x*-axis in the projection fiber, D*xassoc*: diffusivity along the *x*-axis in the association fiber, D*yproj*: diffusivity along the *y*-axis in the projection fiber, D*zassoc*: Diffusivity along the *z*-axis in the association fiber.

In areas with projection fibers, the dominant fibers run along the *z*-axis, while the *x*-axis and *y*-axis are perpendicular. In areas with association fibers, the dominant fibers run along the *y*-axis, and the *x*-axis and *z*-axis are perpendicular. The key difference in water molecule behavior between *x*-axis diffusivity (along the perpendicular axis) in these regions and the perpendicular diffusivity is influenced by the presence of the perivascular space, which impacts how water diffuses in these regions (15). Therefore, the DTI-ALPS index could be associated with the glymphatic system function.

### 2.3 Correlation analysis between DTI-ALPS index and clinical characteristics

We carried out a correlation analysis between the DTI-ALPS index and clinical characteristics including age, CKD stage, hemoglobin, hematocrit, protein, albumin, aspartate aminotransferase, alanine aminotransferase, BUN, creatinine, sodium, potassium, chloride, calcium, phosphate, and total CO_2_ content in patients with CKD.

### 2.4 Statistical analyses

Student’s t-test, Fisher’s exact test, or Pearson correlation were performed using MedCalc® Statistical Software version 20 (MedCalc Software Ltd., Ostend, Belgium). Considering the influence of age when comparing the DTI-ALPS index, additional analysis was conducted by adding age as a co-variate using analysis of covariance (ANCOVA). Statistical significance was set at a two-tailed *p*-value <0.05. Multiple corrections were applied for statistical analysis of structural connectivity (Bonferroni correction, *p* = 0.01 [0.05/5]).

## 3 Results

### 3.1 Clinical characteristics of participants

Table 1 shows the clinical characteristics of these patients. Clinical characteristics did not differ between patients with CKD who had cognitive impairment and who did not.

**Table 1:** Clinical characteristics in patients with CKD and healthy controls.

Clinical data	Patients with CKD (*N* = 56)	Heathy controls (*N* = 38)	*p*-value
Demographic data			
Age, years (SD)	62.9 (8.1)	61.2 (7.5)	0.293
Male, *N* (%)	25 (44.6)	17 (44.7)	0.992
Right-handed, *N* (%)	56 (100)	38 (100)	1.000
Comorbidities			
Diabetes mellitus, *N* (%)	28 (50.0)		
Hypertension, *N* (%)	45 (80.4)		
CKD stage			
2	1 (1.8)		
3	14 (25.0)		
4	2 (3.6)		
5	39 (69.6)		
K-MoCA	21.9 (6.0)		
Laboratory data			
Hemoglobin, g/dL (SD)	10.9 (1.5)		
Hematocrit, % (SD)	33.3 (4.5)		
Protein, g/dL (SD)	6.7 (0.7)		
Albumin, g/dL (SD)	4.3 (3.9)		
Aspartate aminotransferase, U/L (SD)	20.9 (7.2)		
Alanine aminotransferase, U/L (SD)	18.5 (9.6)		
BUN, mg/dL (SD)	47.6 (20.5)		
Creatinine, mg/dL (SD)	6.6 (3.9)		
Sodium, mmol/L (SD)	139.7 (3.0)		
Potassium, mmol/L (SD)	4.6 (0.6)		
Chloride, mmol/L (SD)	101.7 (4.9)		
Calcium, mg/dL (SD)	8.4 (0.9)		
Phosphate, mg/dL (SD)	4.2 (0.9)		
Total CO_2_ contents, mmol/L (SD)	24.1 (4.1)		

Clinical data	CKD patients with CI (*N* = 21)	CKD patients without CI (*N* = 35)	*p*-value
Demographic data			
Age, years (SD)	64.4 (8.2)	62.0 (8.0)	0.282
Male, *N* (%)	6 (28.5)	19 (54.3)	0.063
Comorbidities			
Diabetes mellitus, *N* (%)	8 (38.1)	20 (57.1)	0.171
Hypertension, *N* (%)	18 (85.7)	27 (77.1)	0.439
CKD stage			0.581
2	0 (0.0)	1 (2.9)	
3	7 (33.3)	7 (20.0)	
4	1 (4.8)	1 (2.9)	
5	13 (61.9)	26 (74.2)	
K-MoCA	16.0 (5.6)	25.4 (2.3)	<0.001
Laboratory data			
Hemoglobin, g/dL (SD)	10.7 (1.4)	11.0 (1.6)	0.577
Hematocrit, % (SD)	32.7 (4.5)	33.6 (4.6)	0.495
Protein, g/dL (SD)	6.8 (0.8)	6.6 (0.6)	0.248
Albumin, g/dL (SD)	3.7 (0.5)	4.7 (4.9)	0.399
Aspartate aminotransferase, U/L (SD)	20.5 (7.1)	21.2 (7.3)	0.744
Alanine aminotransferase, U/L (SD)	16.0 (9.0)	20.0 (9.7)	0.125
BUN, mg/dL (SD)	48.7 (20.6)	47.0 (20.8)	0.760
Creatinine, mg/dL (SD)	5.6 (3.8)	7.3 (3.9)	0.130
Sodium, mmol/L (SD)	139.2 (3.8)	140.0 (2.5)	0.323
Potassium, mmol/L (SD)	4.6 (0.6)	4.6 (0.7)	0.924
Chloride, mmol/L (SD)	101.4 (4.8)	101.9 (5.0)	0.751
Calcium, mg/dL (SD)	8.5 (0.5)	8.3 (1.1)	0.398
Phosphate, mg/dL (SD)	4.3 (0.9)	4.2 (0.9)	0.775
Total CO_2_ contents, mmol/L (SD)	24.8 (2.8)	23.8 (4.7)	0.389

CKD, chronic kidney disease; SD, standard deviation; K-MoCA, Korean version of the Montreal Cognitive Assessment; CI, cognitive impairment.

### 3.2 Differences in the structural connectivity between the groups

The measures of structural connectivity differed significantly between patients with CKD and healthy controls (Table 2). The mean clustering coefficient, local efficiency, and small-worldness index in patients with CKD were lower than those in healthy controls (0.125 ± 0.056 vs. 0.167 ± 0.082, *p* = 0.008; 1.191 ± 0.183 vs. 1.525 ± 0.651, *p* = 0.002; 0.090 ± 0.043 vs. 0.143 ± 0.102, *p* = 0.003; respectively).

**Table 2:** Difference of the structural connectivity between the groups.

Structural connectivity measures	Patients with CKD (*N* = 56)	Heathy controls (*N* = 38)	*p*-value
Mean clustering coefficient (SD)	0.125 (0.056)	0.167 (0.082)	0.008
Global efficiency (SD)	0.937 (0.082)	1.074 (0.317)	0.011
Local efficiency (SD)	1.191 (0.183)	1.525 (0.651)	0.002
Characteristic path length (SD)	3.971 (0.388)	4.032 (0.390)	0.460
Small-worldness index (SD)	0.090 (0.043)	0.143 (0.102)	0.003

Structural connectivity measures	CKD patients with CI (*N* = 21)	CKD patients without CI (*N* = 35)	*p*-value
Mean clustering coefficient (SD)	0.124 (0.057)	0.128 (0.055)	0.865
Global efficiency (SD)	0.935 (0.087)	0.940 (0.078)	0.862
Local efficiency (SD)	1.217 (0.187)	1.148 (0.172)	0.268
Characteristic path length (SD)	4.006 (0.365)	3.919 (0.433)	0.477
Small-worldness index (SD)	0.089 (0.044)	0.092 (0.043)	0.855

CKD, chronic kidney disease; SD, standard deviation; CI, cognitive impairment.

However, there were no differences of the structural connectivity between CKD patients with and without cognitive impairment (Table 2).

### 3.3 Differences in the DTI-ALPS index between the groups

**Figure 2:**
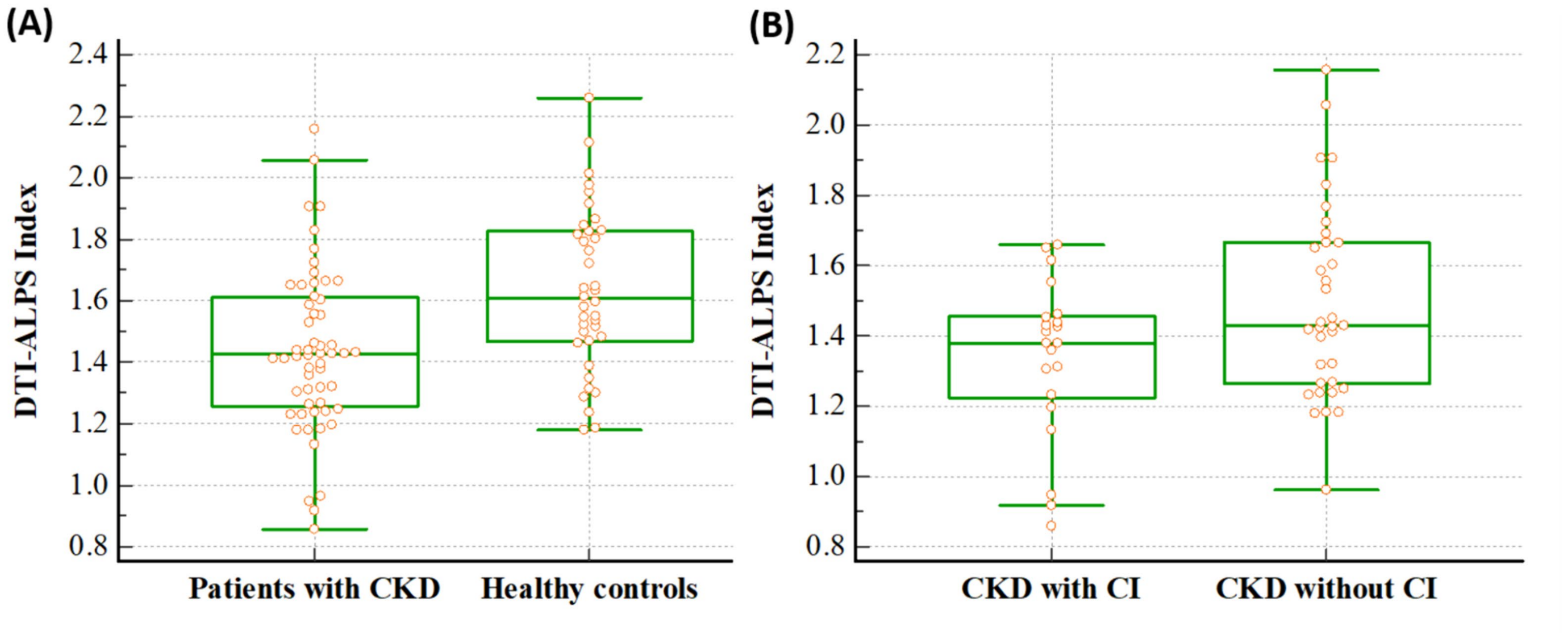
Differences of the DTI-ALPS index between the groups. The figures reveal that DTI-ALPS index in patients with CKD was lower than that in healthy controls (1.436 vs. 1.632, *p* < 0.001) **(A)**, and the DTI-ALPS index in patients with CKD with CI was lower than that in those without CI (1.338 vs. 1.494, *p* = 0.031) **(B)**. A box is drawn from the 25th to 75th percentiles, and a horizontal line is drawn at the median. DTI-ALPS, diffusion tensor image analysis along the perivascular space; CKD, chronic kidney disease; CI, cognitive impairment.

The DTI-ALPS indices differed significantly between patients with CKD and healthy controls. Notably, this index in patients with CKD was lower than that in healthy controls (1.436 ± 0.265 vs. 1.632 ± 0.261, *p* < 0.001) (Figure 2A). In addition, in the ANCOVA analysis with age as co-variate, DTI-ALPS index in patients with CKD was lower than that in healthy controls (*p* < 0.001). When the analysis was conducted only on the remaining 28 patients, excluding those with T2DM, the results remained consistent. The DTI-ALPS index in CKD patients without T2DM was lower than that in healthy controls (1.459 ± 0.146 vs. 1.632 ± 0.261, *p* = 0.002).

Additionally, it differed significantly between CKD patients with and without cognitive impairment, and it was lower in CKD patients with cognitive impairment than in patients without cognitive impairment (1.338 ± 0.226 vs. 1.494 ± 0.272, *p* = 0.031) (Figure 2B). In addition, in the ANCOVA analysis with age as co-variate, DTI-ALPS index in CKD patients with cognitive impairment was lower than that in patients without cognitive impairment (*p* = 0.038).

### 3.4 Correlation analysis between DTI-ALPS index and clinical characteristics

**Figure 3:**
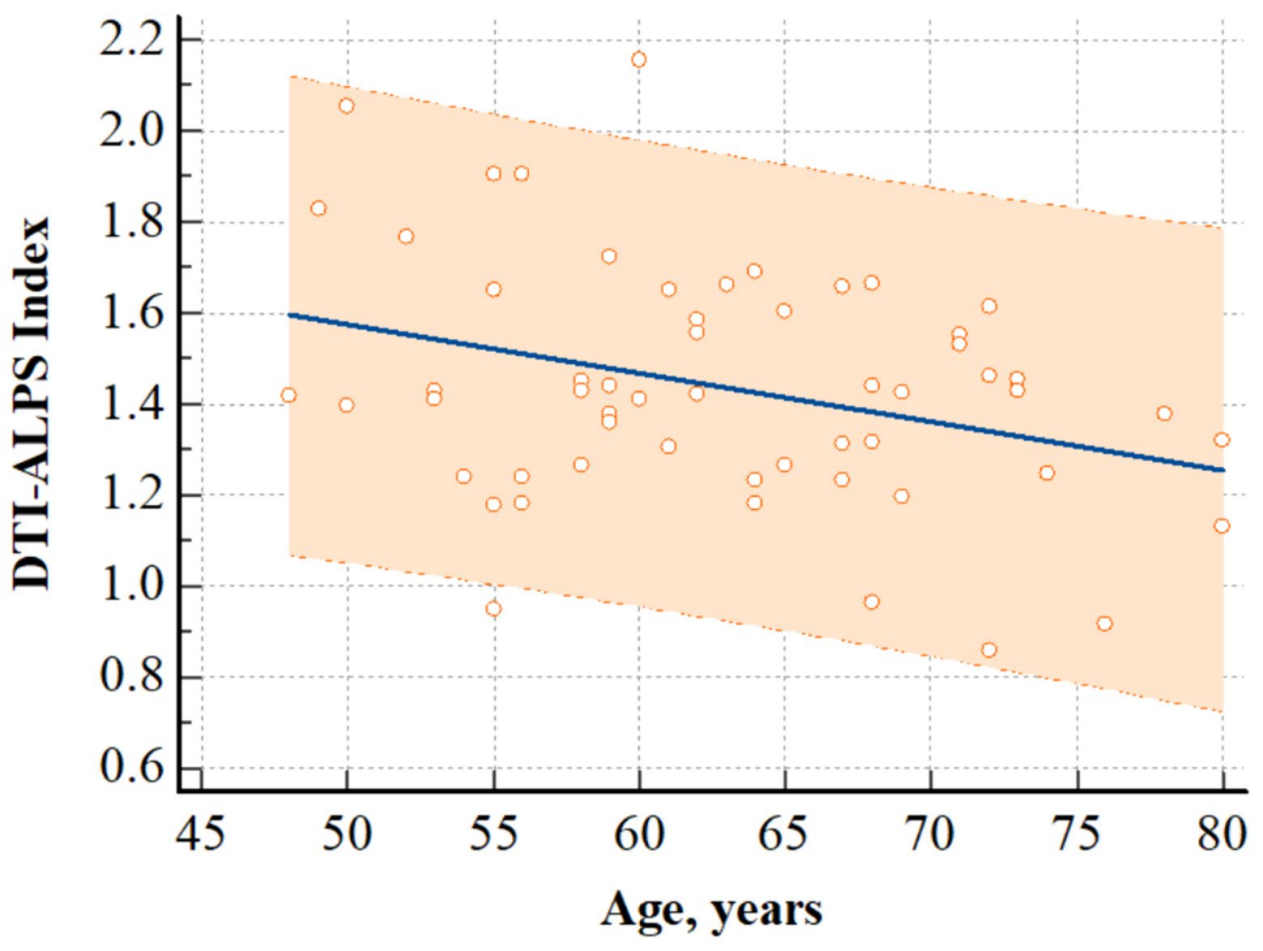
Correlation analysis. The figure shows that there was a significant negative correlation between age and DTI-ALPS index in patients with CKD (*r* = −0.326, *p* = 0.014). Regression line and 95% prediction interval are presented. DTI-ALPS index, diffusion tensor image analysis along the perivascular space; CKD, chronic kidney disease.

In patients with CKD, there was a significant negative correlation between the DTI-ALPS index and age (*r* = −0.326, *p* = 0.014) (Figure 3). However, it did not significantly correlate with other clinical characteristics, including the stage of CKD (*r* = 0.126, *p* = 0.355), and other laboratory data (Hemoglobin, *r* = 0.141, *p* = 0.298; Hematocrit, *r* = 0.140, *p* = 0.305; Albumin, *r* = −0.099, *p* = 0.467; Aspartate aminotransferase, *r* = 0.075, *p* = 0.582; Alanine aminotransferase, *r* = 0.159, *p* = 0.242; BUN, *r* = 0.061, *p* = 0.657; Creatinine, *r* = 0.199, *p* = 0.141; Sodium, *r* = −0.182, *p* = 0.179; Potassium, *r* = −0.186, *p* = 0.169; Chloride, *r* = −0.230, *p* = 0.088; Calcium, *r* = 0.152, *p* = 0.264; Phosphate, *r* = 0.203, *p* = 0.134; Total CO_2_ contents, *r* = 0.010, *p* = 0.940).

## 4 Discussion

We confirmed lower DTI-ALPS index in patients with CKD compared with healthy controls, which may be related with glymphatic system dysfunction. The measures of structural connectivity also differed significantly between patients with CKD and healthy controls. In addition, we showed that cognitive function in patients with CKD was significantly associated with DTI-ALPS index and that the DTI-ALPS index was lower in patients with CKD with cognitive impairment than in those without cognitive impairment. However, there were no differences of the structural connectivity between CKD patients with and without cognitive impairment.

Glymphatic system dysfunction in patients with advanced CKD, notably those with end-stage renal disease (ESRD) has been previously reported (18). The DTI-ALPS index differed between patients with ESRD and healthy controls, and glymphatic system function normalized even after initiating kidney replacement therapy. In this study, we expanded the analysis, including patients with all stages of CKD, and found that lower DTI-ALPS index was also observed in these patients compared to healthy controls, which may be related with glymphatic system dysfunction. Furthermore, the glymphatic system not only acts as an effective waste clearance pathway for the brain but also alters the traditional model of CSF hydrodynamics (28). Theoretically, glymphatic system dysfunction in patients with CKD is related to the decreased expression of AQP-4, including early CKD onset (29). The loss of AQP-4 expression acts to disrupt CSF influx and CSF-ISF turnover. Moreover, this loss contributes to reduced Aβ clearance through the glymphatic system, affecting the increase of neurodegenerative diseases such as Alzheimer’s disease (30). Accordingly, we explored the changes in the DTI-ALPS index in groups with and without cognitive impairment. Notably, in chronic medical disorders, determining the presence or absence of cognitive impairment is important for treatment. The prevalence of cognitive impairment in patients with CKD is 10–40%, which is higher than that in the general population (31). Cognitive impairment is more prevalent in patients with ESRD (32), and it has a multifactorial cause in those with CKD. Traditional cardiovascular risk factors and non-traditional kidney disease-related factors are among the proposed factors. The former is a result of the patients with CKD having higher cardiovascular disease risk factors, including diabetes, hypertension, and dyslipidemia, prevalently associated with cerebrovascular disease (33, 34). Additionally, the related kidney disease results from a steady accumulation of uremic metabolites and dialysis factors, and anemia (35–37). Patients with CKD are characterized by decreased clearance of metabolic waste from the body, and it is thought that cognitive function is reduced due to decreased clearance of metabolic waste from the brain due to decreased glymphatic system function (38). In this study, we found a lower DTI-ALPS index in patients with CKD and cognitive impairment than in those without cognitive impairment.

The DTI-ALPS index, is a valuable neuroimaging biomarker for cognitive function, even after accounting for various covariates and gray matter volumes (39). A previous study showed that a lower DTI-ALPS index and a higher frequency of cognitive impairment were also observed in patients with metabolic syndrome (40). Similarly, cognitive impairment is also associated with a lower DTI-ALPS index in patients with cerebral small vessel disease (41). The association between cognitive impairment and glymphatic system dysfunction has been observed in patients with Alzheimer’s disease. The accumulation of amyloid beta (Aβ) within the brain parenchyma blocks the pathway of interstitial fluid (ISF) drainage, which worsens perivascular clearance (42).

As previously stated, it is plausible that the cognitive impairment in patients with metabolic syndrome is caused by a decrease in waste clearance due to the deposition of various substances. T2DM is the most common metabolic syndrome, and the incidence of dementia has reportedly increased in patients with T2DM (43). Diabetic patients with dementia are referred to as type 3, requiring careful management (44). However, the accumulation of Aβ plaques in the brains of patients with diabetes can affect cognitive function (45). The cognitive impairment in patients with T2DM can lead to an inability to manage hyperglycemia or hypoglycemia, leading to an increased incidence of diabetic complications. Reportedly, cognitive impairment occurs in patients with T2DM owing to glymphatic system dysfunction. Notably, T2DM delays the drainage of ISF into the hippocampus and hypothalamus (46). In this study, half of the patients with CKD also had T2DM. Additionally, we analyzed the differences in the DTI-ALPS index between patients with and without T2DM. However, there was no statistically significant difference in the DTI-ALPS index regardless of the presence of T2DM in this study. This may indicate that hemodynamic changes, such as hypertension, significantly influence glymphatic dysfunction rather than the delay in glymphatic system drainage caused by the accumulation of metabolic substances. In addition to hypertension, increased blood pressure variability is an independent glymphatic system dysfunction risk factor (47, 48).

MoCA is a widely used screening tool designed to assess cognitive impairment. We used the MoCA in this study, because it is particularly useful for detecting cognitive decline in patients with CKD that might not be apparent in other tests, like the mini-mental state exam (MMSE) (49). In patients with CKD, frontal lobe dysfunction is the most common presenting symptom, and the MoCA is better at detecting frontal lobe dysfunction than MMSE (49). However, in this present study, it was difficult to clearly diagnose MCI or dementia because more precise cognitive function tests including ADL were not performed.

We investigated the association between DTI-ALPS index and cognitive function in patients with CKD. However, this study has some limitations. First, we included a small number of patients with CKD at a single center. Second, there was an unequal distribution of patients across different stages of CKD. The inclusion of a similar number of patients at each CKD stage would enhance the reliability of the study results. Third, we excluded the patients with structural brain lesions. However, we did not exclude the patients with microbleed or white matter hyperintensity, which could be related with cognitive function. Fourth, albuminuria also considerably affects cognitive impairment, even more than GFR; however, we were unable to analyze it because of the lack of data. However, in this study, there was a significant disparity in the number of patients across the different stages, which may have influenced the outcomes. Overall, progression to advanced CKD is associated with a higher likelihood of glymphatic system dysfunction, regardless of the cause of CKD. This positively correlates with cognitive impairment; therefore, blood pressure control and correction of cerebrovascular risk factors to prevent CKD progression may help prevent cognitive impairment in CKD.

## 5 Conclusion

We discovered that the DTI-ALPS index was lower in patients with CKD compared to healthy controls, which may be related with glymphatic system dysfunction. Within the CKD group, those with cognitive impairment had a lower DTI-ALPS index than those without, indicating a potential link between the glymphatic system and cognitive function.

## Data Availability

The original contributions presented in the study are included in the article/supplementary material, further inquiries can be directed to the corresponding author.

## References

[1] KDIGO. 2012 clinical practice guideline for the evaluation and management of chronic kidney disease. Kidney Int Suppl. (2013) 3:1. 10.1038/ki.2013.243 23989362

[2] Bikbov B Purcell CA Levey AS Smith M Abdoli A Abebe M. Global, regional, and national burden of chronic kidney disease, 1990–2017: a systematic analysis for the global burden of disease study 2017. Lancet. (2020) 395:709–33. doi: 10.1016/S0140-6736(20)30045-3, PMID: 32061315 PMC7049905

[3] Arnold R Issar T Krishnan AV Pussell BA. Neurological complications in chronic kidney disease. JRSM Cardiovasc Dis. (2016) 5:2048004016677687. doi: 10.1177/2048004016677687, PMID: 27867500 PMC5102165

[4] Bugnicourt J-M Godefroy O Chillon J-M Choukroun G Massy ZA. Cognitive disorders and dementia in CKD: the neglected kidney-brain axis. J Am Soc Nephrol. (2013) 24:353–63. doi: 10.1681/ASN.2012050536 23291474

[5] Madan P Kalra OP Agarwal S Tandon OP. Cognitive impairment in chronic kidney disease. Nephrol Dial Transpl. (2007) 22:440–4. doi: 10.1093/ndt/gfl572 17023495

[6] Mogi M Horiuchi M. Clinical interaction between brain and kidney in small vessel disease. Cardiol Res Pract. (2011) 2011:306189:1–5. doi: 10.4061/2011/306189 21274446 PMC3025374

[7] Madero M Gul A Sarnak MJ. Cognitive function in chronic kidney disease. Semin Dial. (2008) 21:29–37. doi: 10.1111/j.1525-139X.2007.00384.x 18251955

[8] Heo CM Lee WH Park BS Lee YJ Park S Kim YW. Glymphatic dysfunction in patients with end-stage renal disease. Front Neurol. (2022) 12:809438. doi: 10.3389/fneur.2021.809438, PMID: 35145471 PMC8821099

[9] Iliff JJ Wang M Liao Y Plogg BA Peng W Gundersen GA. A paravascular pathway facilitates CSF flow through the brain parenchyma and the clearance of interstitial solutes, including amyloid β. Sci Transl Med. (2012) 4:147ra111. doi: 10.1126/scitranslmed.3003748 PMC3551275 22896675

[10] Nedergaard M Goldman SA. Glymphatic failure as a final common pathway to dementia. Science. (2020) 370:50–6. doi: 10.1126/science.abb8739, PMID: 33004510 PMC8186542

[11] Hablitz LM Nedergaard M. The glymphatic system: a novel component of fundamental neurobiology. J Neurosci. (2021) 41:7698–711. doi: 10.1523/JNEUROSCI.0619-21.2021, PMID: 34526407 PMC8603752

[12] Simard M Arcuino G Takano T Liu QS Nedergaard M. Signaling at the gliovascular interface. J Neurosci. (2003) 23:9254–62. doi: 10.1523/JNEUROSCI.23-27-09254.2003, PMID: 14534260 PMC6740832

[13] Lee DA Park BS Ko J Park SH Lee YJ Kim IH. Glymphatic system dysfunction in temporal lobe epilepsy patients with hippocampal sclerosis. Epilepsia Open. (2022) 7:306–14. doi: 10.1002/epi4.12594 35305294 PMC9159256

[14] Bae YJ Choi BS Kim JM Choi JH Cho SJ Kim JH. Altered glymphatic system in idiopathic normal pressure hydrocephalus. Parkinsonism Relat Disord. (2021) 82:56–60. doi: 10.1016/j.parkreldis.2020.11.009, PMID: 33248394

[15] Taoka T Masutani Y Kawai H Nakane T Matsuoka K Yasuno F. Evaluation of glymphatic system activity with the diffusion MR technique: diffusion tensor image analysis along the perivascular space (DTI-ALPS) in Alzheimer's disease cases. Jpn J Radiol. (2017) 35:172–8. doi: 10.1007/s11604-017-0617-z, PMID: 28197821

[16] Toh CH Siow TY. Glymphatic dysfunction in patients with ischemic stroke. Front Aging Neurosci. (2021) 13:756249. doi: 10.3389/fnagi.2021.756249 34819849 PMC8606520

[17] Heo CM Lee DA Park KM Lee YJ Park S Kim YW. Glymphatic system dysfunction in patients with early chronic kidney disease. Front Neurol. (2022) 13:976089. doi: 10.3389/fneur.2022.976089, PMID: 36003297 PMC9393609

[18] Lee YJ Park KM Heo CM Park S Kim YW Lee D. Changes in the glymphatic system before and after dialysis initiation in patients with end-stage kidney disease. Ren Fail. (2023) 45:2265665. doi: 10.1080/0886022X.2023.2265665, PMID: 37795782 PMC10557553

[19] Yi J Park S Kim YW Park B Jin Lee Y Heo C. Changes in the glymphatic system before and after dialysis initiation in patients with ESRD: SA-PO547. J Am Soc Nephrol. (2023) 34:876–7. doi: 10.1681/ASN.20233411S1876c36757153

[20] Taoka T Naganawa S. Glymphatic imaging using MRI. J Magn Reson Imaging. (2020) 51:11–24. doi: 10.1002/jmri.26892 31423710

[21] Lee YJ Yoon E Park S Kim YW Kim SE Ko J. Alteration of brain connectivity in neurologically asymptomatic patients with chronic kidney disease. Medicine (Baltimore). (2021) 100:e25633. doi: 10.1097/MD.0000000000025633 33879740 PMC8078245

[22] Michna M Kovarova L Valerianova A Malikova H Weichet J Malik J. Review of the structural and functional brain changes associated with chronic kidney disease. Physiol Res. (2020) 69:1013–28. doi: 10.33549/physiolres.934420, PMID: 33129242 PMC8549872

[23] Islam N Hashem R Gad M Brown A Levis B Renoux C. Accuracy of the Montreal cognitive assessment tool for detecting mild cognitive impairment: a systematic review and meta-analysis. Alzheimers Dement. (2023) 19:3235–43. doi: 10.1002/alz.13040, PMID: 36934438

[24] Rolls ET Huang CC Lin CP Feng J Joliot M. Automated anatomical labelling atlas 3. NeuroImage. (2020) 206:116189. doi: 10.1016/j.neuroimage.2019.116189 31521825

[25] Park KM Lee BI Shin KJ Ha SY Park J Kim SE. Pivotal role of subcortical structures as a network hub in focal epilepsy: evidence from graph theoretical analysis based on diffusion-tensor imaging. J Clin Neurol. (2019) 15:68–76. doi: 10.3988/jcn.2019.15.1.68, PMID: 30618219 PMC6325361

[26] Farahani FV Karwowski W Lighthall NR. Application of graph theory for identifying connectivity patterns in human brain networks: a systematic review. Front Neurosci. (2019) 13:585. doi: 10.3389/fnins.2019.00585, PMID: 31249501 PMC6582769

[27] Guye M Bettus G Bartolomei F Cozzone PJ. Graph theoretical analysis of structural and functional connectivity MRI in normal and pathological brain networks. MAGMA. (2010) 23:409–21. doi: 10.1007/s10334-010-0205-z 20349109

[28] Iliff JJ Wang M Zeppenfeld DM Venkataraman A Plog BA Liao Y. Cerebral arterial pulsation drives paravascular CSF–interstitial fluid exchange in the murine brain. J Neurosci. (2013) 33:18190–9. doi: 10.1523/JNEUROSCI.1592-13.2013, PMID: 24227727 PMC3866416

[29] Ampawong S Klincomhum A Likitsuntonwong W Singha O Ketjareon T Panavechkijkul Y. Expression of aquaporin-1, -2 and -4 in mice with a spontaneous mutation leading to hydronephrosis. J Comp Pathol. (2012) 146:332–7. doi: 10.1016/j.jcpa.2011.08.005, PMID: 21945302

[30] Zeppenfeld DM Simon M Haswell JD D’Abreo D Murchison C Quinn JF. Association of perivascular localization of aquaporin-4 with cognition and Alzheimer disease in aging brains. JAMA Neurol. (2017) 74:91–9. doi: 10.1001/jamaneurol.2016.4370, PMID: 27893874

[31] Drew DA Weiner DE Sarnak MJ. Cognitive impairment in CKD: pathophysiology, management, and prevention. Am J Kidney Dis. (2019) 74:782–90. doi: 10.1053/j.ajkd.2019.05.017, PMID: 31378643 PMC7038648

[32] Park KM Heo CM Lee DA Huh H Park S Kim YW. Intrinsic prefrontal functional connectivity according to cognitive impairment in patients with end-stage renal disease. Korean. J Nephrol. (2023). doi: 10.23876/j.krcp.22.291 PMC11615448 37559223

[33] Cheung AK Sarnak MJ Yan G Berkoben M Heyka R Kaufman A. Cardiac diseases in maintenance hemodialysis patients: results of the HEMO Study. Kidney Int. (2004) 65:2380–9. doi: 10.1111/j.1523-1755.2004.00657.x, PMID: 15149351

[34] Drew DA Bhadelia R Tighiouart H Novak V Scott TM Lou KV. Anatomic brain disease in hemodialysis patients: a cross-sectional study. Am J Kidney Dis. (2013) 61:271–8. doi: 10.1053/j.ajkd.2012.08.035, PMID: 23040011 PMC3546146

[35] Meyer TW Hostetter TH. Approaches to uremia. J Am Soc Nephrol. (2014) 25:2151–8. doi: 10.1681/ASN.2013121264, PMID: 24812163 PMC4178448

[36] Eldehni MT McIntyre CW. Are there Neurological Consequences of Recurrent Intradialytic Hypotension? Semin Dial. (2012) 25:253–6. doi: 10.1111/j.1525-139X.2012.01057.x 22353138

[37] Abramson JL Jurkovitz CT Vaccarino V Weintraub WS Mcclellan W. Chronic kidney disease, anemia, and incident stroke in a middle-aged, community-based population: the ARIC Study. Kidney Int. (2003) 64:610–5. doi: 10.1046/j.1523-1755.2003.00109.x, PMID: 12846757

[38] Xu S Wang J Sun K Meng L Qin C Feng R. Cognitive impairment in chronic kidney disease is associated with glymphatic system dysfunction. Kidney Dis (Basel). (2023) 9:384–97. doi: 10.1159/000530635, PMID: 37901711 PMC10601941

[39] Hsu JL Wei YC Toh CH Hsiao IT Lin KJ Yen TC. Magnetic resonance images implicate that glymphatic alterations mediate cognitive dysfunction in Alzheimer disease. Ann Neurol. (2023) 93:164–74. doi: 10.1002/ana.26516 36214568 PMC10091747

[40] Andica C Kamagata K Takabayashi K Kikuta J Kaga H Someya Y. Neuroimaging findings related to glymphatic system alterations in older adults with metabolic syndrome. Neurobiol Dis. (2023) 177:105990. doi: 10.1016/j.nbd.2023.105990, PMID: 36621631

[41] Tang J Zhang M Liu N Xue Y Ren X Huang Q. The association between glymphatic system dysfunction and cognitive impairment in cerebral small vessel disease. Front Aging Neurosci. (2022) 14:916633. doi: 10.3389/fnagi.2022.916633 35813943 PMC9263395

[42] Kamagata K Andica C Takabayashi K Saito Y Taoka T Nozaki H. Association of MRI indices of glymphatic system with amyloid deposition and cognition in mild cognitive impairment and Alzheimer disease. Neurology. (2022) 99:e2648–60. doi: 10.1212/WNL.0000000000201300 36123122 PMC9757870

[43] Gudala K Bansal D Schifano F Bhansali A. Diabetes mellitus and risk of dementia: a meta-analysis of prospective observational studies. J Diab Invest. (2013) 4:640–50. doi: 10.1111/jdi.12087, PMID: 24843720 PMC4020261

[44] Mittal K Katare DP. Shared links between type 2 diabetes mellitus and Alzheimer's disease: a review. Diabetes Metab Syndr Clin Res Rev. (2016) 10:S144–9. doi: 10.1016/j.dsx.2016.01.021 26907971

[45] Prasad S Sajja RK Naik P Cucullo L. Diabetes mellitus and blood-brain barrier dysfunction: an overview. J Pharm. (2014) 2:125. doi: 10.4172/2329-6887.1000125 PMID: 25632404 PMC4306190

[46] Jiang Q Zhang L Ding G Davoodi-Bojd E Li Q Li L. Impairment of the glymphatic system after diabetes. J Cereb Blood Flow Metab. (2017) 37:1326–37. doi: 10.1177/0271678X16654702, PMID: 27306755 PMC5453454

[47] Yang S Qin W Yang L Fan H Li Y Yin J. The relationship between ambulatory blood pressure variability and enlarged perivascular spaces: a cross-sectional study. BMJ Open. (2017) 7:e015719. doi: 10.1136/bmjopen-2016-015719, PMID: 28827244 PMC5724164

[48] Yang S Yuan J Zhang X Fan H Li Y Yin J. Higher ambulatory systolic blood pressure independently associated with enlarged perivascular spaces in basal ganglia. Neurol Res. (2017) 39:787–94. doi: 10.1080/01616412.2017.1324552, PMID: 28475469

[49] Paraizo Mde A Almeida AL Pires LA Abrita RS Crivellari MH Pereira Bdos S. Montreal cognitive assessment (MoCA) screening mild cognitive impairment in patients with chronic kidney disease (CKD) pre-dialysis. J Bras Nefrol. (2016) 38:31–41. doi: 10.5935/0101-2800.20160006, PMID: 27049362

